# The mark of success: The role of vaccine-induced skin scar formation for BCG and smallpox vaccine-associated clinical benefits

**DOI:** 10.1007/s00281-024-01022-9

**Published:** 2024-08-26

**Authors:** Ole Bæk, Frederik Schaltz-Buchholzer, Anita Campbell, Nelly Amenyogbe, James Campbell, Peter Aaby, Christine Stabell Benn, Tobias R. Kollmann

**Affiliations:** 1 University of Copenhagen, Copenhagen, Denmark; 2 University of Southern Denmark, Copenhagen, Denmark; 3 Telethon Kids Institute, Perth, Australia; 4 Dalhousie University, 5980 University Ave #5850, 4th floor Goldbloom Pavilion, Halifax, NS B3K 6R8 Canada; 5 Central Perth Skin Clinic, Perth, Australia; 6 Bandim Health Project, Bissau, Guinea-Bissau

**Keywords:** BCG, Smallpox, Vaccine scar, Dermoscopy, Spatial genomics, Maternal child health

## Abstract

Skin scar formation following Bacille Calmette-Guérin (BCG) or smallpox (*Vaccinia*) vaccination is an established marker of successful vaccination and ‘vaccine take’. Potent pathogen-specific (tuberculosis; smallpox) and pathogen-agnostic (protection from diseases unrelated to the intentionally targeted pathogen) effects of BCG and smallpox vaccines hold significant translational potential. Yet despite their use for centuries, how scar formation occurs and how local skin-based events relate to systemic effects that allow these two vaccines to deliver powerful health promoting effects has not yet been determined. We review here what is known about the events occurring in the skin and place this knowledge in the context of the overall impact of these two vaccines on human health with a particular focus on maternal-child health.

## Introduction

The Smallpox and the Bacille Calmette-Guérin (BCG) vaccine have been among the most commonly administered vaccines in the world [1, 2]. Both vaccines have potent pathogen-specific as well as pathogen-agnostic (protection from diseases unrelated to the intentionally targeted pathogen) effects on the host, including modulation of immunity in pregnant women and their offspring (see section D on maternal-child health below). Both vaccines can be administered via the skin where they lead to scar formation which is considered to reflect ‘vaccine take’ [1, 2]. The molecular mechanisms leading to skin scar formation and their role in creating systemic effects on host protection beyond the skin have not yet been delineated. Following a review of what is known, we outline concrete steps to fill this crucial knowledge gap in order to accelerate building on the clinically proven success of these two potent, proven-effective vaccines.

### A. Smallpox

Prior to the introduction of the smallpox vaccine, smallpox disease accounted for 7% of all annual deaths in the UK, with a case fatality rate of 40% [1–3]. Although the practice of ‘variolation’ (small amounts of pus from a smallpox patient smeared into a break in the skin to confer immunity in an uninfected individual) had been identified long before, deliberate development of the smallpox vaccine from Cowpox virus (which later was changed to the *Vaccinia* virus) by Edward Jenner in 1796 produced the first vaccine [4]. Mandatory smallpox vaccination was introduced in the UK and parts of the USA in newborn children as early as the 1840’s [2, 5], with booster doses recommended for school children and adults [2, 5, 6]. The World Health Organization’s (WHO) campaign to eliminate smallpox succeeded in 1980 [7]. Concerns about bioterrorism and recent rise in the incidence of other Orthopoxvirus infections (Monkeypox, Cowpox, and other zoonotic vaccinia-like viruses; presumed to be linked to the diminished coverage of smallpox immunity due to termination of mandatory mass vaccination) has sparked renewed interest in smallpox vaccines [6, 8–10].

### Pathogen-specific impact of smallpox vaccination

A vesicular lesion followed by subsequent scar formation over the smallpox vaccination site was the original measure of ‘vaccine take’ in an individual prior to immunological testing or clinical trials [11]. Vaccine effectiveness (protection of the population) was measured as reduction of secondary attack rates among vaccinated vs unvaccinated household contacts of cases and was as high as 90–97% in those vaccinated within three years prior to contact exposure [12]. Clinical trials of smallpox vaccination were only conducted in the 1950s more than a century after vaccine roll out. Of note, these initial trials were conducted in neonates [12–16]. Vaccine take was observed in 92% of neonates [14, 17] along with a rise in anti-haemagglutinins against *Vaccinia virus* [18]. A rise of neutralising antibody (Ab) titres by day ten post smallpox vaccination was later established as evidence of vaccine-induced immunity in adult vaccine recipients [19]. Percutaneous administration of smallpox vaccine (scarification) in adults was found to lead to higher neutralizing Ab titres than intradermal or intramuscular administration, and thus became the preferred route of vaccination [20]. Studies in older individuals percutaneously vaccinated > 40 years ago demonstrated persistent neutralising Abs for decades [21–23]. While neutralising Ab titres were shown to correlate well with scar formation [12, 24], they do not appear to affect the progression of smallpox disease once infected, nor are neutralising Ab associated with increased survival among infected individuals [25–27]. This suggests that while antibodies might protect from infection other factors such as cell-mediated immunity and/or innate immunity may be important in protection against smallpox disease [25, 26].

### Pathogen-agnostic impact of smallpox vaccination

Already during the initial introduction of the smallpox vaccine, there were reports of pathogen-agnostic effects ranging from more rapid recovery from skin disorders to decreased risk of contracting other infectious diseases including measles, scarlet fever, whooping cough and syphilis [28–30]. More contemporary cohort studies from Denmark [31, 32] and Guinea-Bissau [32, 33], two countries with very different underlying confounding variables, have confirmed potent pathogen-agnostic benefits of the smallpox vaccine. In Guinea-Bissau prospective studies found that individuals with a smallpox scar indicative of a previous vaccination had better overall survival [34, 35]. This benefit was noted during the 2000s, decades after individuals had been vaccinated with smallpox. Contrary to the pathogen-specific effects that are usually achieved after one dose, the pathogen-agnostic benefits increase with the number of smallpox vaccinations and are stronger in females than in males [34, 35]. In Denmark, the same tendencies were observed in prospective follow-up of the cohorts that experienced the phase-out of smallpox vaccine [32]. In particular, a reduced risk of HIV-1 infection was noted across studies from Denmark and Guinea-Bissau [32, 33]. Combined, the above evidence suggest that smallpox vaccination can confer pathogen-agnostic protection against other infections, similar to what has been observed for other live vaccines [36].

#### Mechanisms of action of the smallpox vaccine (Fig. 1)

As smallpox is a human-only pathogen and the disease has been eradicated in humans, what is known about mechanisms of action is largely derived from animal models using attenuated strains of *Vaccinia virus* (e.g. Modified Vaccinia virus Ankara) that are unable to replicate in human and mammalian cells [37]. In animal models rapid infiltration of innate immune cells (granulocytes in particular) at the site of vaccine administration is followed by local release of Tumour Necrosis factor α (TNF-α), initiating adaptive immune responses in the skin-draining lymph nodes [38]. Vaccination-induced tissue-resident memory T cells home back from the lymph node to not only the site of skin vaccine-administration but populate epithelial surfaces across the body, where they remain detectable for months [39]. These T-cells in the skin can confer protection against infections with unrelated pathogens following smallpox vaccination [39, 40]. And dendritic cells isolated from Modified Vaccinia virus Ankara vaccinated pregnant mice adoptively transferred into naïve neonatal offspring protect them from lethal herpes simplex virus infection [41]. Of interest, protection against sexually transmitted viruses such as simian immune deficiency virus (SIV) in monkeys suggest an increased pathogen-agnostic protection at epithelial surfaces in particular [32, 33, 42, 43]. Specifically, smallpox vaccination induces downregulation of C-C chemokine receptor type 5 (CCR5) on T cells and reduced retroviral replication in peripheral blood mononuclear cells (PBMCs) in vaccinated vs. unvaccinated individuals for up to 3–6 months following vaccination [44, 45]. CCR5 is important for both SIV and HIV to infect their target T cell [46].

### B. BCG

Tuberculosis (TB), caused by *Mycobacterium tuberculosis* (*Mtb*), is responsible for 10 million new cases and 1.5 million deaths each year [47–49]. BCG is currently the only licensed vaccine for TB control, administered to > 120 million people every year [50, 51]. BCG’s inventors, French scientists Albert Calmette and Camille Guérin drew inspiration from smallpox vaccine as they aimed to create a weakened (attenuated) strain of the bovine tuberculosis bacterium that could stimulate an immune response in humans without causing any disease. The first human BCG vaccination was administered orally to a newborn in 1921 [1].

### Pathogen-specific clinical impact of BCG vaccination

In adults, BCG provides only partial protection against TB which wanes over time and varies across populations, with lower efficacy in low- vs. high-income countries [49–54]. Several factors can affect BCG’s efficacy: The number of doses of BCG administered to an individual has been shown to improve TB-specific protection [54–56]. Additionally, the original BCG strain was distributed to various laboratories around the world and over time developed into distinct sub-strains [1]. Despite differences in bacterial morphology and ability to evoke immune responses in animals, it is still unclear if different strains of BCG provide the same degree of protection against TB [57–59]. BCG’s protective efficacy against TB also varies across age, providing far greater protection if given to neonates than adults [59].

### Pathogen-specific mechanisms of action of the BCG vaccine

The mechanism/s through which BCG confers protection against clinical TB are not understood [50, 51]. It is likely that BCG activates multiple protective components of the host, including adaptive and innate immunity as well as other still unknown mechanisms [49, 52]. BCG vaccination does induce an adaptive immune response involving expansion of antigen-specific T and B-cells, leading to formation of *Mtb* specific Th1/Th17 cells along with *Mtb* specific mucosal IgA and humoral IgG responses [49]. Historically, the efficacy of BCG against TB has been assessed by its ability to induce delayed hypersensitivity reactions following intradermal injection of *Mtb*-specific antigens, reflecting active adaptive immune response to *Mtb* driven by interferon-γ releasing helper T cells; however, delayed hypersensitivity reactions do not correlate well with protection against clinical TB [60, 61]. This highlights a significant challenge in TB vaccine research: the lack of identified correlates of protection [49]. Identifying such correlates is crucial for understanding how BCG works, when it works, and for developing more effective TB vaccines that can offer consistent and long-lasting protection [48, 49].

### Pathogen-agnostic clinical impact of BCG vaccination

BCG confers protection not only against TB but also against diseases caused by unrelated pathogens, i.e. pathogen-agnostic effects [36, 62]. BCG’s pathogen-agnostic effects have a long clinical track record as immunotherapy for bladder cancer [50]. Similar to the pathogen-specific effects, BCG’s pathogen-agnostic effects also can vary across age and populations, with more pronounced protection in high infectious disease burden countries [36, 63–66]. In adults BCG may reduce all-cause mortality, risk of respiratory viral infections, and reduce viral titres following challenge with attenuated yellow fever virus [50, 67–69]. Clinical evidence of pathogen-agnostic effects also comes from randomised trials in neonates where BCG can reduce neonatal mortality up to at least 1/3 by preventing deaths from early life infections other than TB, particularly sepsis [36, 62, 66, 70, 71]. Recent clinical trials from Guinea-Bissau found no difference between BCG strains regarding impact on morbidity and mortality [72].

### Pathogen-agnostic mechanisms of action of the BCG vaccine

The mechanisms driving BCG’s pathogen-agnostic effects include changes in innate immunity via ‘trained immunity’ which involves epigenetic and metabolic reprogramming of myeloid cells [73]. The longevity of these innate effects in adults (i.e. innate immune memory) has been attributed to BCG’s effects on the hematopoietic system in the bone marrow, specifically reprogramming hematopoietic stem cells and myeloid progenitor cells that generate long lasting improved innate immune defenses against pulmonary infections [74–78]. Importantly, BCG efficiently stimulates the proliferation and reprogramming of myeloid cells in the bone marrow when delivered intravenously, which go on clear Mtb more efficiently in vitro. This effect is lost when BCG is given intradermally. However, BCG given intradermally to humans induces some epigenetic reprogramming in the hematopoetic lineage resulting in the trained immunity phenotype, as we previously reviewed [79]. Furthermore, in adult mice BCG induces integrated organ immunity that involves adaptive immune cell driven feedback on myeloid as well as epithelial cells that imprint and lead to prolonged and broad innate antiviral resistance [80]. BCG given to murine neonates induces granulocyte colony stimulating factor (G-CSF) within hours, which leads to activation of emergency granulopoiesis with an increase in production of neutrophils in 1–3 days following vaccination [64]. In animal models, this increase in G-CSF and neutrophils are the critical mediators of BCG’s pathogen-agnostic protection from neonatal sepsis [64, 81]. It is possible, that emergency granulopoiesis and trained immunity are on a continuum of the same molecular spectrum, but this has not yet been formally assessed [79]. It is also not known if the mechanisms underpinning trained immunity are the same in neonates and adults.

### BCG’s pathogen-specific protection may relate to its pathogen-agnostic effects

Innate immune memory has also been detected in relation to BCG’s ability to protect from *Mtb* infection itself. Specifically, despite exposure to infectious *Mtb*, previous BCG vaccination enhances the likelihood of remaining interferon gamma release assays (IGRA) negative [62, 82–87]. BCG’s ability to protect from *Mtb* infection via innate (vs. adaptive) immunity has been labelled ‘early clearance’, highlighting the speed with which BCG-induced innate immune mechanisms would have to ‘clear’ mycobacteria before adaptive immune cells ‘detect’ it and an *Mtb*-specific interferon gamma producing T cell response (detected by the IGRA) would be initiated [77, 88, 89]. This early clearance paradigm indicates that BCG’s pathogen-specific protection from *Mtb* infection may in fact relate to BCG’s ability to induce pathogen-agnostic effects. Two studies have assessed BCG’s pathogen-specific together with its pathogen-agnostic impact on public health [90, 91]. Increasing BCG vaccination coverage by vaccinating every child at first medical contact would increase pathogen-specific protection by 11.0% (corresponding to four TB deaths averted per birth cohort of ~ 60,000) and increase pathogen-agnostic protection by 8.1% (corresponding to 392 fewer deaths per birth cohort). Mathematical modelling predicted the cost-effectiveness of such approach to BCG vaccination to be US$ 911 per discounted life-year gained [90]. Additionally, increasing the current global BCG vaccination coverage from 76 to 99%, and scar prevalence among vaccinated infants from 52 to 95% would reduce global infant mortality by > 200,000 deaths/year [91].

### C. Vaccine-induced skin scar formation

#### Skin response to smallpox vaccination (Fig. 1)

Smallpox vaccine was administered through a unique process, termed ‘scarification,’ where it is delivered percutaneously into the dermal layer. Using a bifurcated needle dipped into a reconstituted vial of vaccine, a multiple puncture technique is performed involving repeated jabs (up to 15 times within a 1 cm diameter region) in until a small drop of blood appears [92]. *Vaccinia virus* multiplies and infects the epithelial layers of the skin initially causing redness, then at day 3–5 after vaccination a papule approximately 1 cm in diameter; this is followed by induration, leading to a “Jennerian pustule” by day 5–8 [92]. Crusting and desquamation of the skin overlying the vaccination site occurs by day 14–21, eventually leaving a pitted scar [92]. At the histological level in a non-human primate model of smallpox vaccination, inflammatory cell infiltration and an innate immune response predominate initially, including local recruitment of macrophages and granulocytes as well as monocytoid cells associated with upregulation of TNF pathways [38]. Vaccinia-specific CD8 + T cells are likely generated in skin-draining lymph nodes from where they home back to the skin, including the vaccination site [93] where they can lyse infected target cells and promote resolution of vaccine ‘infection’ [94].

The mode as well as the route of smallpox vaccine delivery can impact scar formation as well as both its pathogen-specific and -agnostic responses. In a rabbit model an immune-protective response becomes detectable within two minutes of scarification. And this protective immune response from local smallpox vaccine ‘infection’ is observed even if *no* vaccine is administered, suggesting the multiple puncture scarification process itself contributes to ‘pathogen-specific/agnostic’ protection [95]. This is likely related to activation of keratinocytes which then act as phagocytes and release preformed chemokines to induce a potent local antiviral state [95]. This scarification response may also be impacted by the skin microbiome, as antibiotic pre-treatment of mice prior to smallpox vaccination reduces the subsequent immune response [96]. Lastly, the importance of the route of smallpox vaccine delivery to enhance pathogen-specific and -agnostic effects has been highlighted in animal models [43, 97]. In a non-human primate model, Modified Vaccinia Ankara expressing Env (i.e. a model SIV-vaccine) led to neutrophil infiltration and activation, resulting in protection against an experimental intravaginal SIV challenge only in intradermally but not intramuscularly vaccinated animals [43, 98, 99]. Events in the skin thus impact both pathogen-specific as well as -agnostic protection following smallpox vaccination [93].

#### Skin response to BCG vaccination (Fig. 1)

Intradermal administration of the standard adult or newborn BCG dose leaves a visible but transient blanching ‘wheal’ with a diameter of 4–5 mm (following the newborn dose of 0.05 ml) and 7–10 mm (following the adult dose of 0.1 ml). In BCG-naïve adults this is usually followed within 1–2 days by formation of a red indurated area of 5–15 mm and formation of a crust, which around 3–4 weeks softens at the centre, often rupturing with pustular discharge continuing for weeks to months. After 2–5 months, the crust that eventually formed falls off, leaving a flat 7- to 10 mm scar that remains stable for years [100, 101]. In newborns, a similar process occurs but with a slight initial delay, with the skin reaction starting 2–4 weeks post vaccination [102]. This reaction also leads to ulcer formation in the newborn that heals spontaneously over the following weeks [101]. Histological examination by hematoxylin-eosin stain of skin biopsies taken near the site of BCG administration reveals lymphocytic infiltrations with granuloma formation 7 days after BCG vaccination, which is slowly replaced over weeks by inflammatory infiltrates composed primarily of epithelioid macrophages and lymphocytes in well-formed non-caseating granulomas [103]. Skin biopsy specimens taken directly from the centre of the BCG administration site via punch biopsies allow detection of BCG by quantitative PCR (qPCR) or culture [104–107]. This assessment revealed correlation between pre-challenge immune status and recovery of BCG bacteria from the biopsy at the group (BCG-vaccinated vs. naïve) as well at the individual participant level. Variation of individual protection from BCG was prominent at earlier (< 14 days) time points, suggesting variation in innate immunity as a possible underlying cause.

**Fig. 1:**
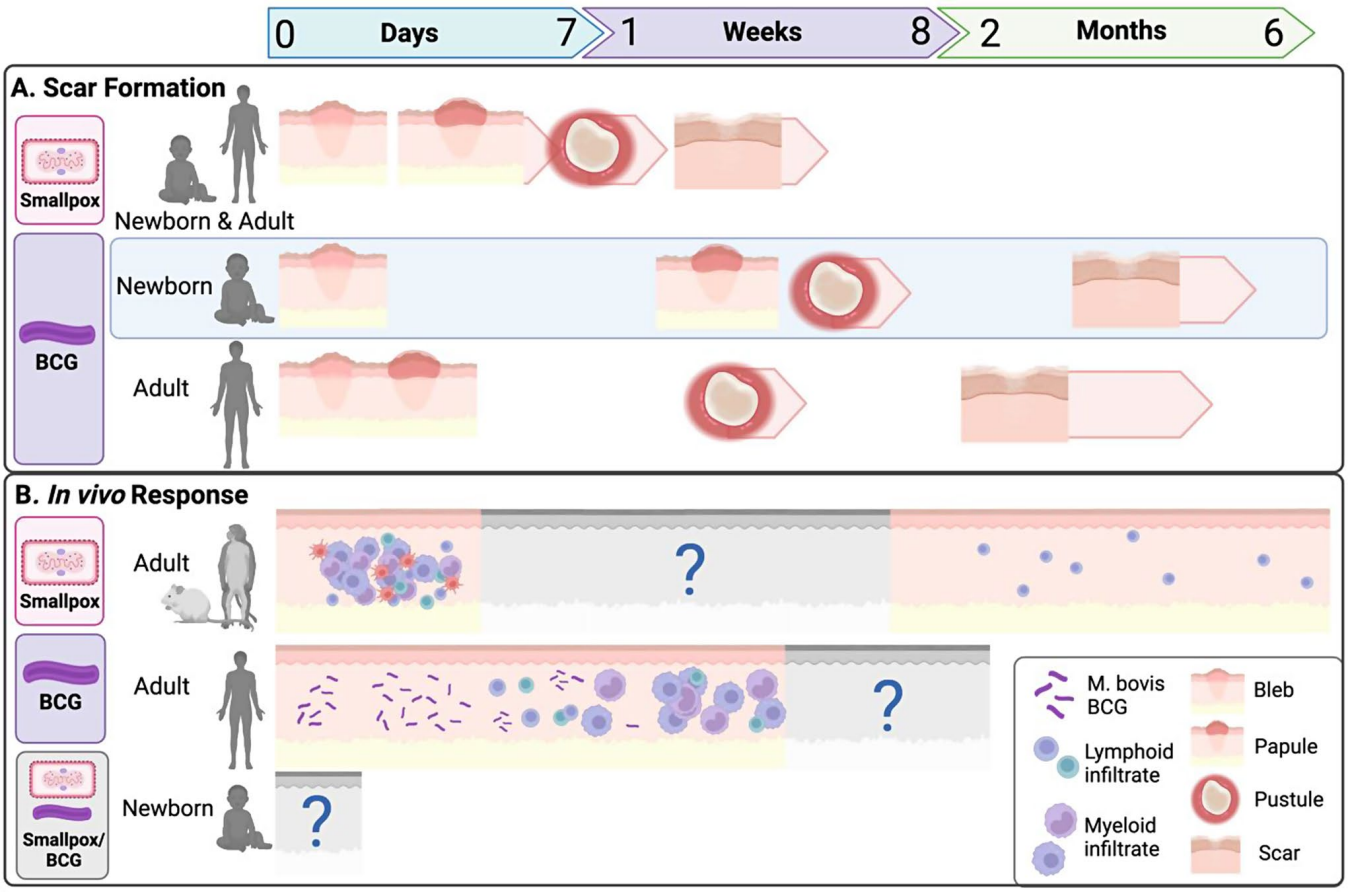
Timeline of scar formation and in vivo responses to Smallpox and BCG vaccination among newborns and adults. **A**. Timing of scar formation following smallpox vaccination is similar among newborns and adults, with a papule occurring within 3–5 days of vaccination, pustules evident within a week, and crusting/desquamification between 14 and 21 days. For BCG vaccination, different kinetics have been observed for newborn vs. adult vaccination, whereby papules are observed 1–2 days post vaccination for adults and 2–4 weeks following vaccination for newborns, followed by more rapid progression from pustule to scar formation in adults compared to newborns. **B**. Known in vivo responses to Smallpox and BCG vaccination in the skin, whereby evidence from animal models demonstrates early myeloid and lymphoid infiltrate 24–72 h post vaccinia vaccination in primate models. Vaccinia-specific CD8 T cells were found in vaccinated mice up to 6 months following vaccination. For BCG vaccination, evidence from adult human studies identifies M. bovis BCG in the skin from 0 to 14 days following vaccination, with low to no levels by day 14. Lymphoid infiltrate is observed from 1–4 weeks post vaccination, followed by myeloid infiltrate from 2–4 weeks post vaccination. Grey areas indicate lack of data at these time points, including any animal or human data for newborn responses

### D. Relevance of vaccine-induced scar for maternal-child healh

Epidemiological data suggest that a maternal BCG or smallpox scar, as evidence of past successful vaccination of the mother, can impact pregnancy outcomes as well as neonatal protection from infection.

#### Impact of smallpox vaccination on maternal and child health

*a)* Maternal smallpox disease is a serious infection with a significant maternal mortality rate (~ 60% vs. 10–30% in unvaccinated pregnant vs. non-pregnant women, and 26% vs. 2% in vaccinated pregnant vs. non-pregnant women) [25, 26, 108]. Furthermore, over two thirds of women infected during pregnancy suffer foetal loss through either stillbirth or spontaneous abortion, and over half of all live-born neonates die within the first 2 weeks of life if the mother suffered smallpox disease during pregnancy [25, 26, 109]. Given this risk profile, in smallpox endemic regions pregnancy was viewed as a definite indication for smallpox vaccination during pregnancy rather than a contraindication [26]. However, smallpox vaccination prior to but not during pregnancy was recommended in non-endemic regions [109]. A recent meta-analysis of smallpox vaccination included 12,201 vaccinated pregnant women and found no association between smallpox vaccination during pregnancy and spontaneous abortion, preterm birth, or stillbirth, with instead a trend towards possible reduction of preterm birth in smallpox vaccinated pregnant women. Thus, smallpox vaccination in pregnancy appears safe if not beneficial for pregnant women [109]. This data supports the current recommendation that in the event of a bioterrorism attack pregnant women should be vaccinated with the smallpox vaccine [109, 110].

*b) Neonatal Health.* Historical data from India identified the lowest incidence of smallpox disease in newborns born to vaccinated mothers [26]. Given the rise in incidence of smallpox disease in these children starting from 1 month of life onward, maternally-transferred protection appears to wane from one month [26]. Based on these findings, India, during endemic times, recommended that irrespective of maternal vaccination status, every newborn be vaccinated immediately after birth or at least before the end of the first month of life [26]. Neonatal vaccinations in any setting (UK, US, India) had an excellent safety profile and displayed vaccine take (scar formation) rates similar to adult recipients [2, 5, 26]. The importance of scar formation in relation to protection following newborn vaccination was also identified in India, where 2,500 children were vaccinated at birth and followed up for 2 years [26]. There was a higher mortality rate in those exposed to smallpox in the household without scar formation (3/4, 75%) compared to no deaths in those exposed but who had formed a scar (0/3, 0%). This data suggests that newborn smallpox vaccination confers protective immunity if a scar is formed [26].

#### Impact of BCG vaccination on maternal and child health

*a) Maternal Health.* TB presents a significant health challenge for pregnant women [111, 112]. While the immune changes that accompany pregnancy likely contribute to this heightened risk [113, 114], the extent to which these immunological alterations during pregnancy amplify the severity of TB in pregnant women remains unclear as the treatment outcomes for clinical TB during pregnancy are comparable to those observed in non-pregnant women [115, 116]. However, the occurrence of clinical TB during pregnancy is associated with a higher likelihood of adverse pregnancy outcomes, including stillbirth, preeclampsia, and premature birth [117, 118]. BCG vaccination is recommended by the WHO prior to (as opposed to during) pregnancy, not because of known adverse events of BCG in pregnancy, but because risks of BCG administered during pregnancy have not been sufficiently assessed [119].

*b) Neonatal Health.* Neonatal TB, while rare, poses marked health risks for newborns. Congenital TB infection has infrequently been reported and infants can acquire TB post-birth through droplet exposure [115]. Whether pathogen-specific immunity against *MtB* is vertically transferred from the mother to the newborn and offers any protection against clinical TB in early life, remains unknown because the majority of infants in TB endemic regions receive BCG at birth. The role of maternal BCG vaccination and scar status has however been investigated, with findings suggesting a positive impact on pathogen-agnostic protection if a maternal BCG scar is present. A trial in Denmark showed that in infants born to mothers with a history of BCG vaccination, randomisation to neonatal BCG vaccination was associated with a 35% (6–55%) reduction in the risk of hospital admission due to infectious diseases when compared to unvaccinated newborns born to mothers with a BCG vaccination history, whereas there was no effect of neonatal BCG vaccination in children of BCG-unvaccinated mothers [120, 121]. This finding of a particularly positive effect of neonatal BCG in children whose mothers were primed with BCG was corroborated in two studies from Guinea-Bissau [122, 123]. For instance, while a maternal BCG scar was not a determinant for development of a scar in the newborn following neonatal BCG vaccination, among the children, having a BCG scar was associated with a 66% reduced all-cause mortality if the mother had a BCG scar but only 8% (95% CI, -83–53%) if the mother had no BCG scar [122]. And for vulnerable newborns (e.g. preterm or low birth weight), enhanced in-hospital survival has been associated with the presence of a maternal BCG scar, with particular benefit to male offspring [124]. Furthermore, the presence of a maternal BCG scar may improve pregnancy outcomes [125]. Specifically, in a prospective observational study from Guinea-Bissau involving 1320 pregnant women, absence of a maternal BCG scar was associated with a 29% (-1% to -68%) increase in the risk of adverse pregnancy outcomes (namely increased risk for miscarriage, stillbirths and early neonatal deaths) [125].

#### Mechanisms potentially connecting vaccine-induced maternal scar for BCG or smallpox vaccination with clinical benefits for the pregnancy and newborn (Fig. 2)

the presence of a smallpox or BCG vaccine-induced maternal scar indicates induction of immunity in the mother. Transfer of maternal immunity from mother to newborn could directly impact pregnancy outcomes, protection of the neonate and/or modulate the neonatal immune response to BCG or smallpox vaccination [126]. There is a lack of understanding however regarding the mechanism through which a BCG- or smallpox-vaccinated mother with a scar might transmit additional protection to her offspring and this requires further investigation,

*a) Transfer of Maternal Adaptive Immunity*: Transfer of adaptive immunity (such as antibodies) from mother to newborn can modulate offspring response to infection and/or vaccines [126]. BCG-specific antibodies may also play a role in protection from TB in early life as the rise in incidence of disseminated TB coincides with the trough in anti-BCG antibody levels, i.e. from 6 months to 3 years of life [127, 128]. Additionally in two uncontrolled studies, smallpox-unvaccinated infants (*n* = 949) born to women who had previously received smallpox vaccine found newborns and infants had anti-haemagglutinins against *Vaccinia* in their blood early in life, presumably obtained via transplacental passage of immunoglobulin G (IgG) [129, 130]. Although not assessed for BCG or smallpox vaccines, transfer of antigen-specific T cells across the placenta can lead to microchimerism, where maternal T-cells might impact immune regulation in the foetus and neonate/infant [131–133]. Finally, adaptive cellular immunity could also be transferred via colostrum from mother to newborn [134].

*b) Transfer of Maternal Innate Immunity*: Transfer of innate immune modulators across the placenta is well established [135]. BCG can induce G-CSF and possibly other growth factors [64, 136] which are relevant for pathogen-agnostic protection [64, 79, 81]. G-CSF is known to cross the placenta, stimulate foetal granulopoiesis and improve neonatal survival for premature newborns. Furthermore, G-CSF promotes trophoblast growth and placental metabolism, which could lead to a reduction of adverse pregnancy outcomes [137]. Although this has never been assessed, maternal BCG-induced G-CSF could potentially explain both reductions in adverse pregnancy outcomes as well as increased neonatal survival. Finally, BCG like beta-glucan could possibly impact inducing transfer of protective innate immune molecules from mother to fetus via oocytes [138].

*c) Transfer of the vaccine*: The placenta does not block *Vaccinia* virus from crossing to the foetus, i.e. maternal smallpox vaccination could induce foetal immune responses to smallpox [26]. Similarly, BCG can systemically disseminate within hours of administration, suggesting that administration of BCG during pregnancy could immunize the foetus [139]. Given the potential for long persistence of BCG in the host, this could possibly occur years to decades after BCG vaccination. Specifically, BCG administered to the mother, even decades earlier, has been identified on the foetal side of the placenta, in the foetus and the newborn as a cell wall deficient, atypical but culturable L-form of BCG [140–144]. It is also possible that microbial antigens could be transferred via breastmilk, inducing or modulating immune responses in the newborn [145].

**Fig. 2:**
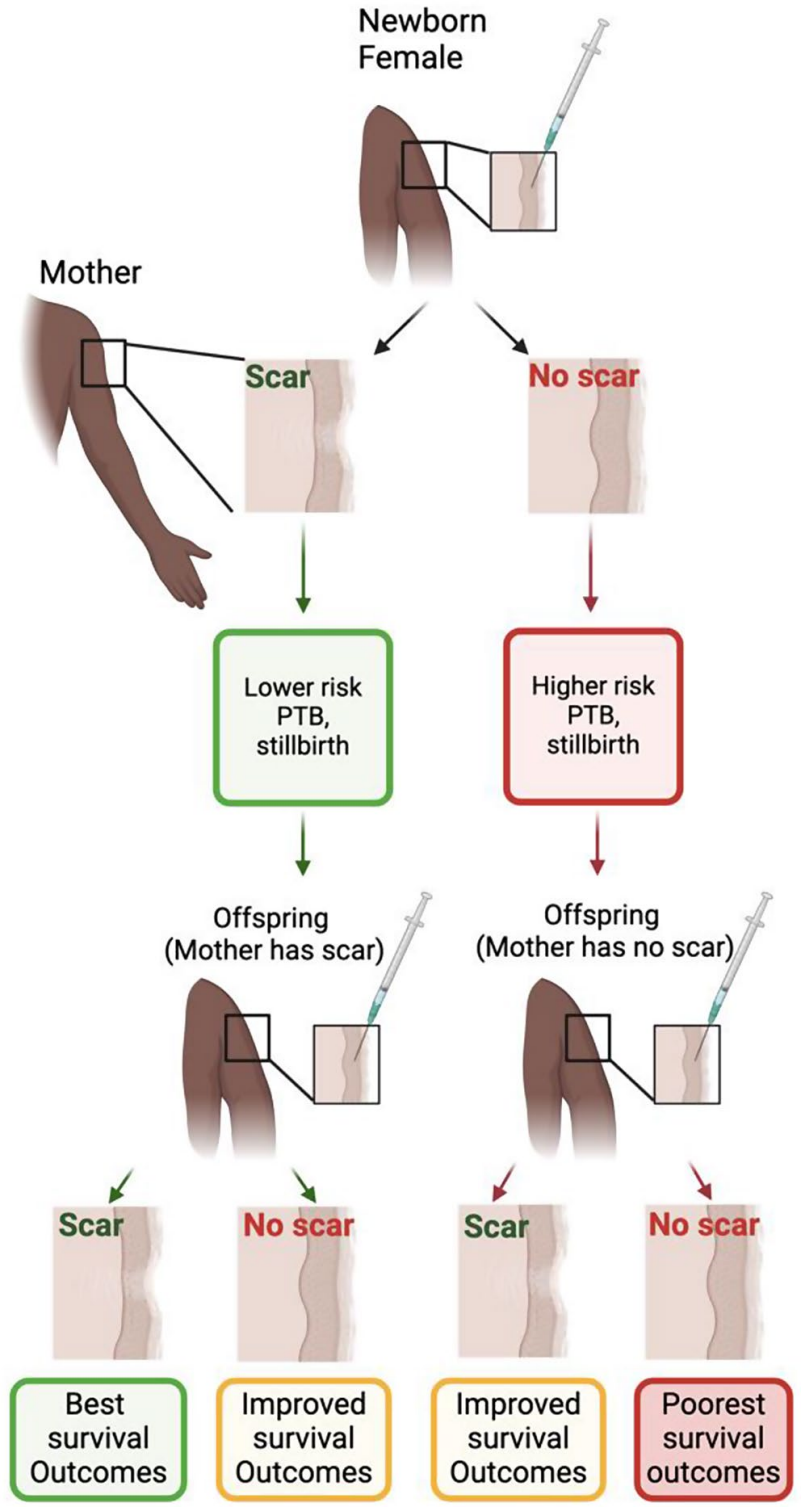
Associations between maternal and newborn BCG scar and health outcomes. Mothers who have a BCG scar (indicative of successful BCG vaccination as newborns) are less likely to deliver pre-term or stillborn children. Among infants born to mothers with a scar, those with a scar have the best outcomes, but those without a scar still have improved outcomes compared to infants with no scar whose mothers also did not have a scar. Infants with a scar whose mothers did not have a scar still have improved outcomes. Collectively, infant survival outcomes are improved if the mother has a BCG scar, independent of their scar status. However, BCG vaccination is still beneficial for infants even if the mothers do not have a scar. The mechanism of these observed outcomes is yet to be defined

### E. Filling the current knowledge gap

The smallpox and BCG vaccines are the oldest and among the most commonly administered vaccines in human history; yet we do not sufficiently understand their mechanisms of action in regard to the many clinical benefits they provide, including both pathogen-specific (protection acute smallpox and Mtb infection and -agnostic (other infectious diseases) effects. Current findings are mostly based on epidemiological data. Many of those were observational studies, and though the findings have largely been corroborated in randomised trials, one should remain careful not to over-interpret findings. Despite this limitation, these epidemiological data stem from robust sample sizes allowing for adjustment for likely host confounders. Further, immunological studies require careful design to recapitulate complex human phenomena, thus on their own also have limitations. Identifying congruence between epidemiological studies and appropriately designed immunological studies is thus most likely to deliver actionable insight. Encouragingly, recent technological advances now allow establishing complementary functional evidence. Specifically, connecting objective clinical evaluation of the macroscopic skin changes following vaccination via dermoscopy with the modern molecular tools of spatial genomics conducted on minimally invasive tissue biopsies and analyses of immune signatures in blood samples collected simultaneously may help fill this crucial knowledge gap.

#### Dermoscopy

Dermoscopy is a non-invasive in vivo method of skin examination, allowing the operator to view patterns and structures from the surface down to events deeper in the skin [146]. It relies on polarized light to minimize skin surface reflectance making it more transparent and allowing the operator to view subsurface structures to the level of the papillary dermis. This facilitates examination of the skin beyond what is visible to the naked eye. Traditionally used to aid in diagnosis of cutaneous malignancy, dermoscopy is increasingly used to examine inflammatory skin conditions (inflammoscopy) [147]. It has further been validated for use in both fair Fitzpatrick skin types and in coloured skin [148]. Furthermore, dermoscopy has been validated as a tool for assessment of scar vascularity and pigmentation [146, 149]. A dermoscope is a mobile and relatively cost-effective device which can easily be attached to a camera or smart phone to allow photographs to be taken of the skin that can then be objectively analyzed via image analysis, as is already done for the diagnosis of both inflammatory and malignant skin lesions [147]. To our knowledge, dermoscopy has not yet been deployed to assess scar formation following smallpox or BCG vaccination. We would expect that in the immediate post-vaccination stages, dermoscopy would reveal inflammatory changes such as redness as well as proliferation or engorgement of superficial vessels, followed by early scale formation as well as formation of a pustule beneath the skin, with ulceration detected long before a clinically apparent scar. Other dermoscopic features that may be observed during scar formation include changes to surface texture, scar thickness, hyper- or hypo-pigmentation, changes to the patterns of pigmentation, and the development of crystal structures within the scar tissue (using polarised dermoscopy) [146]. Together, these patterns may be able to predict early development of scar formation, allowing not only comparison of subjects who develop vaccine-induced scar vs those who do not, but also facilitating correlation of early molecular changes (see below) with final outcome to deliver potential early biomarkers of successful ‘vaccine take’.

#### Tissue is the issue

In order to determine the mechanisms of action for the smallpox and BCG vaccine, modern tools such as spatial genomics (*Nature Methods’* method of the year in 2020 [150]) enable assessing the tissue response to vaccination on the single cell level and within the 3-dimensional context of tissue biopsy samples. These biopsies are no more invasive to obtain than a standard large bore needle routinely used for blood draws. This approach would allow for the highly multiplexed characterization of mechanistic networks required to dissect the complex cell-cell interactive processes in tissues in relation to their systemic benefits for health [151, 152].

Generating insights into the causal mechanistic chain from skin to placenta to fetus and newborn infant offers to fill knowledge gaps preventing efforts to identify interventions that could reduce adverse pregnancy outcomes and neonatal sepsis in particular [66, 70, 71, 153–155]. While smallpox vaccination is currently not recommended, the potential to reduce child mortality by optimization of BCG vaccination, so that almost all BCG vaccinations end with the formation of a scar, might avert hundreds of thousands of deaths and disabilities every year [91]. Furthermore, past clinical trials have relied on the presence of a visible BCG scar to verify previous BCG vaccination. It has therefore not been possible to unequivocally determine if BCG administration induces protection from TB disease, infection with *Mtb*, or pathogen-agnostic protection in those who are vaccinated without a scar versus those who developed a scar after vaccination [83, 156]. Likewise, even though BCG-induced scar formation and scar size correlate with protection in terms of reduced all-cause mortality in infants [102, 157], pathogen-specific response to drug therapy of TB disease [83] as well as *Mtb*-specific adaptive T-cell mediated immunity, scar presence or size does not correlate with BCG-induced protection from TB disease [48, 49, 103, 158]. Yet, while the tuberculin skin response does not correlate well with pathogen-specific protection against Mtb, it does correlate with pathogen-agnostic protection [159, 160]. These complex, unresolved relationships between clinical outcomes, scar formation, and BCG vaccination limits investigation of the true protective effect of the vaccine, independent of the scar, in an era where randomised trials of BCG versus placebo to newborns in low-income settings pose ethical challenges [83, 156]. Given the current knowledge void, scar formation following either BCG or smallpox vaccination is interpreted as the consequences of a presumed immune response to local insults only. Variance concrete molecular cause-effect chains that connect the local reaction to clinical outcomes have therefore been understudied. To allow progress, we now need to fill the mechanistic knowledge gap around scar formation and local immune responses in relation to systemic effects by identifying biomarkers that reliably predict outcome.

## Data Availability

There is no additional data associated with this manuscript.
